# Multi-Adaptive Strategies-Based Higher-Order Quantum Genetic Algorithm for Agile Remote Sensing Satellite Scheduling Problem

**DOI:** 10.3390/s24154938

**Published:** 2024-07-30

**Authors:** Xiaohan Sun, Yuan Ren, Linghui Yu

**Affiliations:** 1 Department of Aerospace Engineering and Technology, Space Engineering University, Beijing 101416, China; 2 DFH Satellite Co., Ltd., Beijing 100094, China; linghuiyu@126.com; 3 Department of Basic Course, Space Engineering University, Beijing 101416, China; renyuan_823@aliyun.com

**Keywords:** adaptive strategies, agile remote sensing satellite scheduling, global optimization, large-scale tasks, quantum genetic algorithm

## Abstract

The agile remote sensing satellite scheduling problem (ARSSSP) for large-scale tasks needs to simultaneously address the difficulties of complex constraints and a huge solution space. Taking inspiration from the quantum genetic algorithm (QGA), a multi-adaptive strategies-based higher-order quantum genetic algorithm (MAS-HOQGA) is proposed for solving the agile remote sensing satellites scheduling problem in this paper. In order to adapt to the requirements of engineering applications, this study combines the total task number and the total task priority as the optimization goal of the scheduling scheme. Firstly, we comprehensively considered the time-dependent characteristics of agile remote sensing satellites, attitude maneuverability, energy balance, and data storage constraints and established a satellite scheduling model that integrates multiple constraints. Then, quantum register operators, adaptive evolution operations, and adaptive mutation transfer operations were introduced to ensure global optimization while reducing time consumption. Finally, this paper demonstrated, through computational experiments, that the MAS-HOQGA exhibits high computational efficiency and excellent global optimization ability in the scheduling process of agile remote sensing satellites for large-scale tasks, while effectively avoiding the problem that the traditional QGA has, namely low solution efficiency and the tendency to easily fall into local optima. This method can be considered for application to the engineering practice of agile remote sensing satellite scheduling for large-scale tasks.

## 1. Introduction

**Figure 1:**
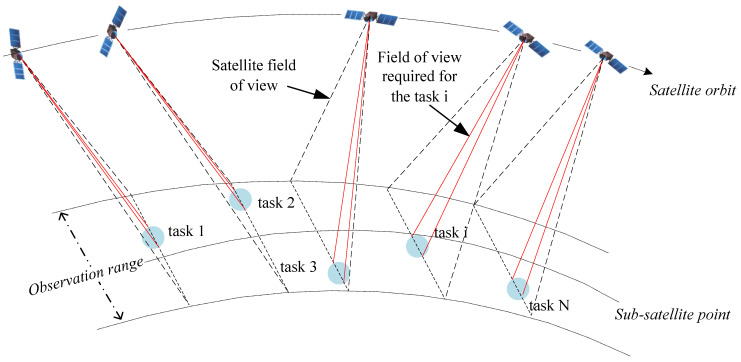
Imaging schematic diagram of agile remote sensing satellite.

Remote sensing satellites are particularly effective tools for observing and analyzing earth’s resources and environment and play an irreplaceable role in earth resource exploration, natural disaster prevention, and environmental protection [1]. Compared to traditional non-agile remote sensing satellites, as shown in Figure 1, the advantage of agile remote sensing satellites lies in their flexible and fast maneuverability, which not only greatly enhances the observation capabilities of satellites but also leads to a significant increase in the complexity of solving satellite execution plans [2].

Before the rapid development of agile remote sensing satellites, many scholars had conducted research over a long period on the scheduling problem of non-agile remote sensing satellites. Previous research mainly focused on explaining the concept of satellite scheduling. Due to the lack of large-scale task sets, satellite scheduling models were often simplified [3,4,5,6]. Abramson [7] considered satellite constraints, including satellite lateral sway and storage and introducing an integer linear programming method for finding a solution. Bianchesi [8] takes the COSMO SkyMed constellation as the research object, with the goal of maximizing the number of images obtained, and adopts a heuristic construction algorithm to solve the satellite scheduling problem, but does not introduce enough real constraints. Florio [9] considered the mobility constraints of satellites in his research, while also considering the energy and storage capabilities of satellites as constraints. Lemaître [10] took into account the strong maneuverability of agile remote sensing satellites and considered the satellite’s maneuverability model in detail, but simplified the processing by incorporating additional constraints for the satellite. Reference [11] treated the decomposition of regional targets as a type of constraint for satellites. Reference [12] proposed and to some extent solved the resource matching problem based on conventional observation tasks and emergency observation tasks, with task efficiency as the global optimization objective in the scheduling process. Peng’s research also focuses on this aspect [13]. Xu [14] focused on satellite observation time window constraints and energy constraints, simplifying other constraints. Reference [15] focused on addressing the observation time dependency in the scheduling process of agile remote sensing satellites in his research, but simplified other constraints. The algorithm proposed by Reference [16] can effectively improve the real-time performance of computation, but its feasibility for large-scale tasks has not been verified. Du’s research focuses on multi-star mission scheduling, and his proposed method is beneficial for the efficiency of large-scale task allocation, but does not consider the single-star constraint problem in detail [17].

Starting from the end of the twentieth century, intelligent heuristic optimization algorithms have been widely used in the field of satellite mission planning and scheduling due to their good optimization capabilities, such as the ant colony algorithm [18,19], the particle swarm optimization algorithm [20], the genetic algorithm [21,22], the improved genetic algorithm [23,24,25,26,27], the simulated annealing algorithm [28,29,30], the taboo search algorithm [31,32,33,34,35], and evolutionary algorithms [36,37]. The complexity of agile remote sensing satellite mission scheduling based on large-scale tasks has increased exponentially, and intelligent heuristic optimization algorithms have shown the disadvantage of low solution efficiency during the solution process. Therefore, traditional algorithms are in urgent need of further innovation and development. In recent years, artificial intelligence has been applied in various fields. Many scholars have combined artificial intelligence algorithms with satellite scheduling problems and conducted a series of studies [38,39,40,41,42,43]. In addition, many scholars have applied physics-informed deep learning approaches to many fields and proved the algorithm’s superior optimization ability [44]. Similarly, it is feasible to apply this method to the field of satellite mission scheduling. The biggest feature of this type of method is that it can improve the timeliness of satellite execution plans while achieving global optimization. However, these methods are limited by the performance of on-board computers and have certain limitations in the current field of engineering applications.

Currently, in the engineering practice process of satellite mission scheduling, how to efficiently and reliably generate satellite task execution plans is a key issue that urgently needs to be solved. Narayanan [45] integrated the ideas of genetic algorithms and quantum theory and demonstrated that this algorithm has a significant effect on improving search capability and computational speed. Silveira [46] innovatively proposed a quantum-inspired evolutionary algorithm and demonstrated its effectiveness through sorting problems. Nowotniak [47] introduced the concept of high-order on the basis of a quantum genetic algorithm and proved that this method has significant effects on improving computational speed.

In our study, we propose a novel MAS-HOQGA to solve the agile remote sensing satellite scheduling problem. First, taking into account the constraints, including the time-dependent characteristics of agile remote sensing satellites, attitude maneuverability, payload observation capability, data transmission resources, storage capability, and energy balance, a refined satellite constraint model with comprehensive multiple constraints was established. The scheduling objective function was established based on the comprehensive value derived from the number of tasks and their priorities. Then, on the basis of the traditional QGA, the quantum register operator, the adaptive evolution strategy, and the adaptive mutation transfer strategy are introduced. The proposed MAS-HOQGA is used to solve the agile satellite scheduling problem. 

Compared to traditional QGA, the quantum chromosome in this paper is composed of quantum register operators, ensuring that the proposed algorithm has advantages in both individual measurement and updates. Quantum registers can reduce the time complexity of individual measurements and updates, thereby improving algorithm performance. In addition, the adaptive evolution strategy and adaptive mutation transfer strategy introduced in this paper are beneficial for improving the convergence speed of the algorithm and preventing it from falling into local optima.

**Figure 2:**
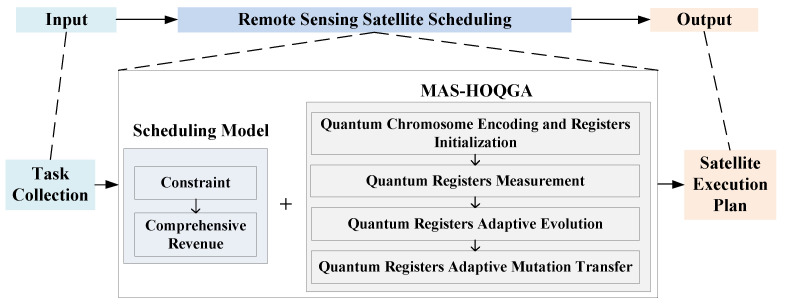
Framework diagram of the paper.

The experimental results show that the MAS-HOQGA has achieved significant improvements in both comprehensive revenue and algorithm running time for scheduling results compared with the QGA. Figure 2 shows the framework of the research content of this paper.

Specifically, the specific work and innovation of the paper are as follows:

(1) Considered the agile remote sensing satellite mission scheduling problem for large-scale tasks scenarios.

(2) Established a comprehensive multi-constraint satellite scheduling model and described the optimization objective function based on the total task number and the total task priorities.

(3) A higher-order QGA based on multi-adaptive strategies was proposed.

(4) The proposed algorithm shows excellent computing power and global optimization capabilities in the field of agile remote sensing satellite mission scheduling.

This paper is divided into five parts. After the introduction, Section 2 introduces the establishment process of the multi-constraint refined satellite scheduling model. Section 3 introduces MAS-HOQGA, including quantum individual encoding and quantum register initialization, the measurement and adaptive evolution strategies of quantum register operators, and the adaptive mutation transfer strategy of quantum registers. In Section 4, the comprehensive revenue and algorithm running time of the MAS-HOQGA and QGA for scheduling tasks of different scales are compared, and the impact of the probability amplitude adjustment parameter setting on the results is analyzed. The final section presents the conclusions of this paper.

## 2. Refined Satellite Scheduling Model with Multi-Constraints

The establishment of a satellite mission scheduling model needs to consider the usage constraints of the satellite and the requirements of its various subsystems. In this section, the relevant parameters in the satellite scheduling model are defined, and the constraints of each subsystem in the satellite scheduling model are given.

### 2.1. Parameter Definition

The satellite properties are defined as follows:

$M_{max}$: satellite storage maximum;

$m_{ut}$: satellite payload data rate;

$E_{max}$: satellite energy maximum;

$e_{ut}^{p}$: the energy consumption per unit of time during satellite payload operation;

$e_{ut}^{d}$: the energy consumption per unit of time during satellite maneuvering;

$A_{ut}$: the angle at which a satellite can maneuver its orientation per unit of time;

Fov: the satellite payload field of view angle.

The task properties are defined as follows:

$X=\left\{\begin{array}{cc} \begin{array}{ccc} X_{1}, & X_{2}, & \ldots \end{array}, & \begin{array}{ccc} X_{i}, & \ldots , & X_{N} \end{array} \end{array}\right\}$: the collection of tasks, where *N* is the total number included in X, and $X_{i}$ is the *i*-th observation task.

$X_{i}=\left\{\begin{array}{cc} \begin{array}{cc} \begin{array}{cc} {id}_{i}, & {prio}_{i}, \end{array} & \begin{array}{cc} {lat}_{i}, & {lon}_{i}, \end{array} \end{array} & \begin{array}{cc} \begin{array}{cc} {alt}_{i}, & {dur}_{i}, \end{array} & \begin{array}{cc} {obs}_{i,bgn}, & {obs}_{i,end} \end{array} \end{array} \end{array}\right\}$: ${id}_{i}$ is the task ID, ${prio}_{i}$ is the task priority, ${lat}_{i}$ is the task latitude, ${lon}_{i}$ is the task longitude, ${alt}_{i}$ is the task altitude, ${dur}_{i}$ is the task observation duration, ${obs}_{i,bgn}$ is the task observation began time, and ${obs}_{i,end}$ is the task observation end time.

The task window properties are defined as follows:

${WIN}_{i}=\left\{\begin{array}{cc} \begin{array}{cc} {win}_{1,i}, & {win}_{2,i}, \end{array} & \begin{array}{cc} \cdots , & {win}_{m,i}, \end{array} \end{array}\right\}$: the observation windows set of task $X_{i}$, *m* is the total number of observation windows corresponding to the mission $X_{i}$, where ${win}_{m,i}=\left\{\begin{array}{ccc} {Orb}_{m.i}, & {win}_{m,i}^{Bgn}, & {win}_{m,i}^{End} \end{array}\right\}$, ${Orb}_{m.i}$ is the number of cycles in which the observation window is located, ${win}_{m,i}^{Bgn}$ is the beginning time of the window, and ${win}_{m,i}^{End}$ is the end time of the window.

### 2.2. Scheduling Model Constraints

#### 2.2.1. Payload Constraints

Based on the working capacity of the payload, specify the maximum working time within one orbit of the payload and require that the working time of each orbit of the payload be less than the maximum working time.

(1) $$\sum_{i=0}^{k}{Payload}_{{dur}_{i}}^{{orb}_{j}}<{Payload\_T}_{max}^{orb}$$

where *k* is the number of observation tasks arranged within the *j*-th orbital cycle, ${Payload}_{{dur}_{i}}^{{orb}_{j}}$ is the observation duration of the *i*-th payload mission arranged within the orbital cycle *j*, and ${Payload\_T}_{max}^{orb}$ is the maximum working duration of the payload within one orbital cycle.

#### 2.2.2. Maneuverability Constraints

**Figure 3:**
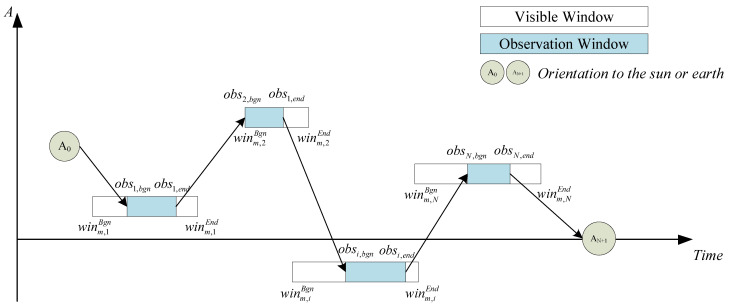
Schematic diagram showing constraints for agile earth observation satellite maneuverability.

The time interval between two observation tasks needs to be greater than the fastest maneuvering time of the satellite, as shown in Figure 3.

(2) $${obs}_{i,bgn}-{obs}_{i-1,end}\geq \frac{A_{ut}}{\left(A_{i}-A_{i-1}\right)},i\in \left(2,N\right)$$

#### 2.2.3. Energy Constraints

During the operation of the satellite, it is necessary to always ensure the satellite energy balance; that is, the energy consumed by the satellite during operation should be less than the maximum energy consumption.

(3) $$\sum_{i=1,j=1}^{N,N+1}\left({e_{ut}^{p}dur}_{i}+e_{ut}^{d}\frac{A_{j}-A_{j-1}}{\left(A_{ut}\right)}\right)\leq E_{max},i\in \left(1,N\right),j\in \left(1,N+1\right)$$

*N* is the count of successfully arranged observation tasks, *N* + 1 is the number of attitude maneuvers arranged, $A_{0}$ is the initial attitude of the satellite, and $A_{N+1}$ is the attitude that the satellite needs to maintain after completing all planned tasks.

#### 2.2.4. Data Storage Constraints

According to the design requirements for satellite data storage, it is required that the data generated by the payload be promptly arranged to the ground, and they cannot exceed the satellite storage maximum.

(4) $$\sum_{i=1}^{N}m_{ut}{dur}_{i}\leq M_{max}$$

#### 2.2.5. Comprehensive Revenue Scheduling Function

In the actual engineering scheduling process, satellite users hope that the satellite can observe more tasks on one hand, and on the other hand, they also hope that the satellite can prioritize completing higher priority tasks. This paper considers both the number of observation tasks and their corresponding priorities. A comprehensive scheduling revenue function is established as the objective function for the scheduling solution.

(5) $$Rev=\omega_{1}\sum_{i=1}^{N}y_{i}+\omega_{2}\sum_{i=1}^{N}y_{i}{pro}_{i}$$

(6) $$y_{i}=\left\{\begin{array}{l} 1,\;the\;task\;X_{i}\;is\;observed\\ 0,\;the\;task\;X_{i}\;is\;not\;observed \end{array}\right.$$

where $\omega_{1}$ represents the revenue weight corresponding to the number of observation missions, $\omega_{2}$ represents the revenue weight corresponding to the priority of the observation missions, and $y_{i}$ serves as a decision variable.

## 3. Solution Methodology

### 3.1. Quantum Chromosome Encoding and Register Initialization

In quantum computing, quantum genes are composed of qubits, with $\left|\left.0\right\rangle \right.$ and $\left|\left.1\right\rangle \right.$ being the basic elements that make up a qubit. The qubits $\left|\left.q\right\rangle \right.$ are represented as follows:

(7) $$\left|\left.q\right\rangle \right.=\alpha \left|\left.0\right\rangle +\beta \left|\left.1\right\rangle \right.\right.$$

In the equation above, the probability amplitudes of $\left|\left.0\right\rangle \right.$ and $\left|\left.1\right\rangle \right.$ being α and β, respectively, the probability of obtaining 0 for this gene locus after measurement is ${\left|\alpha \right|}^{2},$ and the probability of obtaining 1 for this gene locus after measurement is ${\left|\beta \right|}^{2}$. 

For a scheduling scheme consisting of N observation tasks, the encoding of quantum chromosome $\left|\left.q_{\left[X\right]}\right\rangle \right.$ can be represented as

(8) $$\left|\left.q_{\left[X\right]}\right\rangle \right.=\left[\begin{array}{ccc} \begin{array}{cc} \alpha_{1} & \alpha_{2}\\ \beta_{1} & \beta_{2} \end{array} & \begin{array}{cc} \cdots & \alpha_{m}\\ \cdots & \beta_{m} \end{array} & \begin{array}{cc} \cdots & \alpha_{N}\\ \cdots & \beta_{N} \end{array} \end{array}\right]$$

In the above-mentioned quantum chromosome encoding, each qubit of the quantum chromosome is represented by a set of independent binary genes, and there is no connection between qubits. Such a quantum chromosome is called a quantum chromosome of order-1. The difference between higher-order quantum chromosomes and quantum chromosomes of order-1 is that the qubits of higher-order quantum chromosomes will affect each other, and the scope of influence depends on the order of the higher-order quantum chromosomes. Taking quantum chromosome of order-2 as an example, two consecutive qubits constitute an independent quantum register, and each quantum register is a superposition state composed of any linear combination of four basic states $\left|\left.00\right\rangle \right.$, $\left|\left.01\right\rangle \right.$, $\left|\left.10\right\rangle ,\right.$ and $\left|\left.11\right\rangle \right.$.

(9) $$\left|\left.R\right\rangle \right.=\alpha_{0}\left|\left.00\right\rangle +\alpha_{1}\left|\left.01\right\rangle \right.\right.+\alpha_{2}\left|\left.10\right\rangle \right.+\alpha_{3}\left|\left.11\right\rangle \right.$$

In the above equation, the probability amplitudes of $\left|\left.00\right\rangle \right.$, $\left|\left.01\right\rangle \right.$, $\left|\left.10\right\rangle ,\right.$ and $\left|\left.11\right\rangle \right.$ are $\alpha_{0}$, $\alpha_{1}$, $\alpha_{2},$ and $\alpha_{3}$, respectively. The probability of obtaining $\left|\left.00\right\rangle \right.$ for this register after measurement is ${\left|\alpha_{0}\right|}^{2}$, the probability of obtaining $\left|\left.01\right\rangle \right.$ for this register after measurement is ${\left|\alpha_{1}\right|}^{2}$, the probability of obtaining $\left|\left.10\right\rangle \right.$ for this register after measurement is ${\left|\alpha_{2}\right|}^{2}$, the probability of obtaining $\left|\left.11\right\rangle \right.$ for this register after measurement is ${\left|\alpha_{3}\right|}^{2}$.

The probability amplitudes meet the normalization requirements and are all initialized to $\frac{1}{2}$. This operation can ensure that the initial state of each individual in the initial population is consistent, thereby ensuring that each individual in the initial solution has the same probability of being selected.

(10) $${\left|\alpha_{0}\right|}^{2}+{\left|\alpha_{1}\right|}^{2}+{\left|\alpha_{2}\right|}^{2}+{\left|\alpha_{3}\right|}^{2}=1$$

$${\left|\frac{1}{2}\right|}^{2}+{\left|\frac{1}{2}\right|}^{2}+{\left|\frac{1}{2}\right|}^{2}+{\left|\frac{1}{2}\right|}^{2}=1$$

For a scheduling scheme consisting of N observation tasks, the encoding of a quantum chromosome of order-2 $\left|\left.R_{\left[X\right]}\right\rangle \right.$ can be represented as follows:

(11) $$\;\left|\left.R_{\left[X\right]}\right\rangle \right.=\left[\begin{array}{cc} \begin{array}{ccc} \begin{array}{c} \begin{array}{c} \alpha_{0}^{1}\\ \alpha_{1}^{1} \end{array}\\ \begin{array}{c} \alpha_{2}^{1}\\ \alpha_{3}^{1} \end{array} \end{array} & \begin{array}{c} \begin{array}{c} \alpha_{0}^{2}\\ \alpha_{1}^{2} \end{array}\\ \begin{array}{c} \alpha_{2}^{2}\\ \alpha_{3}^{2} \end{array} \end{array} & \begin{array}{c} \begin{array}{c} \ldots \\ \cdots \end{array}\\ \begin{array}{c} \cdots \\ \cdots \end{array} \end{array} \end{array} & \begin{array}{ccc} \begin{array}{c} \begin{array}{c} \alpha_{0}^{m}\\ \alpha_{1}^{m} \end{array}\\ \begin{array}{c} \alpha_{2}^{m}\\ \alpha_{3}^{m} \end{array} \end{array} & \begin{array}{c} \begin{array}{c} \ldots \\ \cdots \end{array}\\ \begin{array}{c} \cdots \\ \cdots \end{array} \end{array} & \begin{array}{c} \begin{array}{c} \alpha_{0}^{2/N}\\ \alpha_{1}^{2/N} \end{array}\\ \begin{array}{c} \alpha_{2}^{2/N}\\ \alpha_{3}^{2/N} \end{array} \end{array} \end{array} \end{array}\right]$$

**Figure 4:**
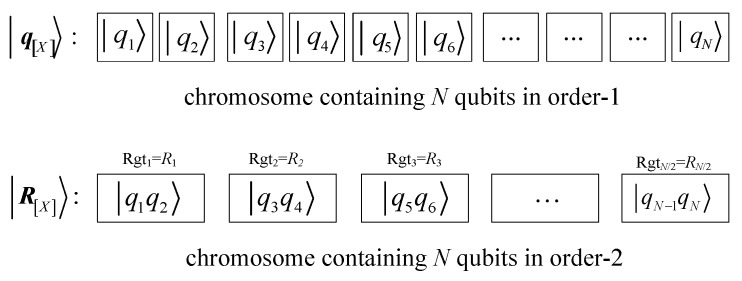
Quantum chromosomes composed of independent qubits and quantum registers.

The schematic diagram of a quantum chromosome composed of independent qubits and quantum registers is shown in Figure 4.

### 3.2. Quantum Registers Measurement

Quantum chromosomes require a step involving a measurement operator to collapse each quantum register from a superposition state into a definite state. For a quantum chromosome of order-2, it is necessary to collapse each quantum register composed of two consecutive qubits, from a superposition state composed of any linear combination of the four basic states, into a definite measurement value. The measurement function returns the measurement value, while recording the index corresponding to the basic states of $\left|\left.00\right\rangle \right.$, $\left|\left.01\right\rangle \right.$, $\left|\left.10\right\rangle \right.,$ and $\left|\left.11\right\rangle \right.$. Algorithm 1 is the quantum registers measurement strategy pseudocode.
**Algorithm 1** Quantum registers measurement1: **Input:** $\left|\left.R_{i,j}\right\rangle \right.=\alpha_{i,0}^{j}\left|\left.00\right\rangle \right.+\alpha_{i,1}^{j}\left|\left.01\right\rangle \right.+\alpha_{i,2}^{j}\left|\left.10\right\rangle \right.+\alpha_{i,3}^{j}\left|\left.11\right\rangle \right.$2: **Output:** $M\left(\left|\left.R_{i,j}\right\rangle \right.\right)$, *probability amplitude index* in {0, 1, 2, 3}3: r ← uniformly random number from [0, 1]4:   **if** $r<{\left|\alpha_{i,0}^{j}\right|}^{2}$**then**5:       $M\left(\left|\left.R_{i,j}\right\rangle \right.\right)$ ← 006:       *probability amplitude index* ← 07:   **else if** $r<{\left|\alpha_{i,0}^{j}\right|}^{2}+{\left|\alpha_{i,1}^{j}\right|}^{2}$ **then**8:      $M\left(\left|\left.R_{i,j}\right\rangle \right.\right)$ ← 019:      *probability amplitude index* ← 110:   **else if** $r<{\left|\alpha_{i,0}^{j}\right|}^{2}+{\left|\alpha_{i,1}^{j}\right|}^{2}+{\left|\alpha_{i,2}^{j}\right|}^{2}$**then**11:      $M\left(\left|\left.R_{i,j}\right\rangle \right.\right)$ ← 1012:      *probability amplitude index* ← 213:   **else**14:      $M\left(\left|\left.R_{i,j}\right\rangle \right.\right)$ ← 1115:      *probability amplitude index* ← 316:   **end if**

### 3.3. Adaptive Evolution for Quantum Registers

In quantum genetic algorithms, quantum gate rotation is a way for each generation of individuals to evolve. The basic principle for quantum gate rotation is to ensure that the current quantum individual evolves towards the optimal quantum individual. The evolution of higher-order quantum chromosomes also ensures that each quantum individual evolves toward the optimal quantum individual. The difference lies in that the evolution method of quantum registers is not the same as that of traditional qubits. specifically, the basic evolution principle of quantum registers is to reduce the probability amplitude of the basic state that does not corresponding to the probability amplitude index.

**Figure 5:**
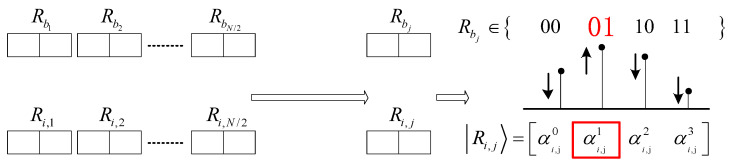
Quantum registers evolution operator.

Let quantum register *j* ($j\in \left(1,2,\ldots ,N/2\right)$) of the quantum individual *i* ($i\in \left(1,2,\ldots ,Q\right)$) currently be represented as $\left|\left.R_{i,j}\right\rangle \right.$. Figure 5 shows the schematic diagram of the quantum register evolution operator. $\left|\left.R_{b_{j}}\right\rangle \right.$ is the best individual from the previous generation. The length of the vertical line segment represents the probability of obtaining the corresponding state of the quantum register after measurement of $\left|\left.R_{i,j}\right\rangle \right.$. According to the logic of quantum register evolution, for the four states of the *j*-th register of the current individual $\left|\left.R_{i,j}\right\rangle \right.,$ which needs to evolve, except for the probability amplitude corresponding to $\alpha_{i,j}^{1},$ which needs to be increased, the probability amplitude of the other three states needs to be reduced, where μ∈ (0,1) is the probability amplitude adjustment parameter.

In the progress of quantum evolution, the probability amplitude adjustment parameter will influence the converge result. When the value of μ is too large, premature convergence may occur, while when the value of μ is too small, the convergence rate is too slow. Accelerating the speed of quantum individual evolution can effectively improve computational efficiency. This paper sets the probability amplitude adjustment parameter to a dynamic adaptive value that changes with the evolution state. The probability amplitude adjustment parameter is determined by the fitness of the currently quantum register to be evolved, the fitness of the optimal quantum register.

(12) $$\mu =\frac{{Fitness}_{opt}-{Fitness}_{cur}}{{Fitness}_{opt}}$$

Algorithm 2 presents the pseudocode for the adaptive evolution strategy of quantum registers.
**Algorithm 2** Quantum register evolution1: **Input:** $\left|\left.R_{i,j}\right\rangle \right.$, $\left|\left.R_{b}\right\rangle \right.$2: **Output:** $\left|\left.R_{i,j}\right\rangle \prime \right.$3: **for** *i* = 1: *Q*4:  **for** *j* = 1: *N*/25:  *R_i_*_, *j*_′= [0 0 0 0]6:  *bestindex* ←probability amplitude index in $\left|\left.R_{b}\right\rangle \right.$7:  **sum ←** 08:   **for** *tmpindex* = 0: 39:    **if** *tmpindex*≠ *bestindex* **then**10:     *R_i_*_, *j*_′ [*tmpindex*] ← *µ* ·*R_i_*_, *j*_ [*tmpindex*]11:     *sum* ← *sum* + (*R_i_*_, *j*_′ [*tmpindex*])^2^12:    **end if**13:   **end for**14:   *R_i_*_, *j*_′ [*bestindex*] ← $\sqrt{1-sum}$15:  $\left|\left.R_{i,j}\right\rangle \prime \right.$ ←*R_i_*_, *j*_′16:  **end for**17: **end for**

### 3.4. Adaptive Mutation Transfer in Quantum Registers

Quantum mutation operations are usually not considered in traditional quantum genetic algorithms because, compared to genetic algorithms, QGA contains richer encoding information, ensuring population diversity. However, the evolution principle states that each quantum individual evolves toward the optimal quantum individual, which may lead to algorithm premature convergence and falling into local optima. This paper innovatively proposes an adaptive quantum mutation transfer strategy which increases gene diversity and improves search ability through this operation, preventing the process from falling into local optima.

The adaptive mutation transfer strategy of quantum registers requires the introduction of mutation transfer probability parameters, which can affect gene diversity and algorithm efficiency. Algorithm 3 presents the pseudocode for the adaptive mutation transfer strategy applied to quantum registers.
**Algorithm 3** Adaptive mutation transfer for quantum registers1: **Input:** *P*_m_, *R_i_*_, *j*_2: **Output:** *R_i_*_, *j*_3: m ←random integer from [1, *N*/2]4: n ←random integer from {0, 1, 2, 3}5: r ← uniformly random number from [0, 1]6: **for** *i* in 0, . . . , *Q* − 1 **do**7:  **if** r < *P*_m_ **then**8:   *tmp* **=** $\alpha_{i,n}^{m}$9:   **if** n = 0 **then**10:    $\alpha_{i,0}^{m}$ ←$\alpha_{i,1}^{m}$11:    $\alpha_{i,1}^{m}$ ← *tmp*12:   **else if** n = 1 **then**13:    $\alpha_{i,1}^{m}$ ←$\alpha_{i,0}^{m}$14:    $\alpha_{i,0}^{m}$ ← *tmp*15:   **else if** n = 2 **then**16:    $\alpha_{i,2}^{m}$ ←$\alpha_{i,3}^{m}$17:    $\alpha_{i,3}^{m}$ ← *tmp*18:   **else**19:    $\alpha_{i,3}^{m}$ ← $\alpha_{i,2}^{m}$20:     $\alpha_{i,2}^{m}$ ← *tmp*21:   **end if**22:   **end if**23: **end for**

**Figure 6:**
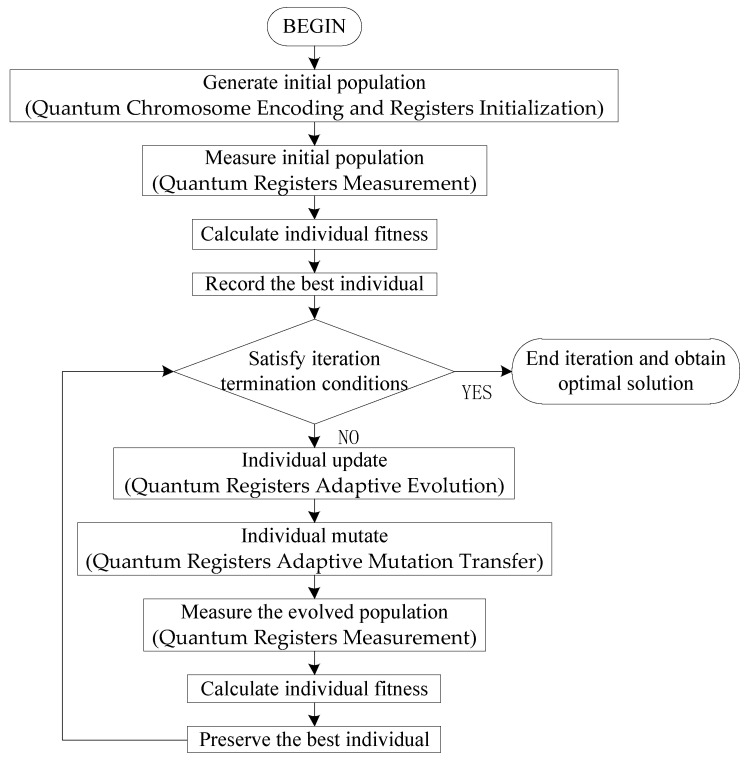
The MAS-HOQGA implementation flow chart.

Figure 6 shows the implementation flow chart for the MAS-HOQGA.

## 4. Computational Experiment

This section first introduces the experimental environment, scenario parameters, and algorithm parameter settings. Then, the experimental results are presented and some analyses are given.

### 4.1. Experimental Environment

The environment for the computational experiment was implemented on an Intel Core I7 CPU (2.80 GHz) with 16 GB of RAM, running the Win10 operating system on a 64-bit architecture. MATLAB R2021b was used for coding.

### 4.2. Parameter Settings

Table 1 shows the orbit parameter information.

The satellite attributes are as follows: the payload field of the view angle is set to 45°, the minimum solar altitude angle is set to 5°, the maximum maneuvering angular velocity is set to 1.3 °/s, the maximum maneuvering angular acceleration is set to 0.01 °/s^2^. The satellite attributes mentioned above are typical parameters in realistic satellite operations.

The order of the quantum chromosome was set to 2. The satellite attributes of individuals’ population scale number Q was set to 50. The satellite attributes of the number of iterative evolutions were set to 100. The mutation transfer probability parameter *P*_m_ = 0.05. The revenue weight corresponding to the number of observation missions $\omega_{1}$ = 0.35. The revenue weight corresponding to the priority of observation missions $\omega_{2}$ = 0.65.

**Figure 7:**
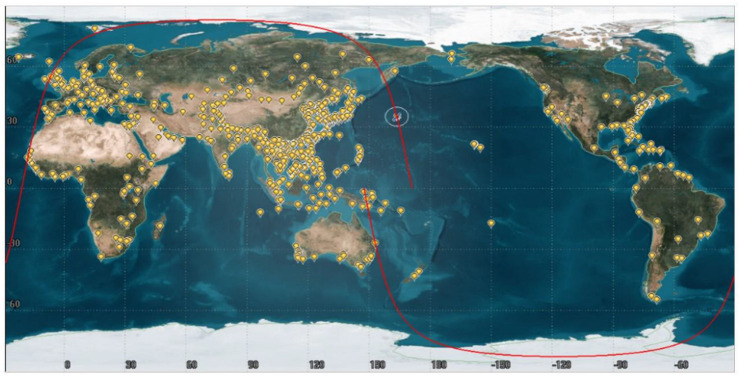
Observation target distribution map.

Target information includes the following: within 80° north and south latitude, the number of 226, 426, and 626 observation targets are randomly generated. The distribution of observation targets is shown in Figure 7, where the red line represents the satellite trajectory.

### 4.3. Experimental Results and Analyses

#### 4.3.1. Comparison of Results between MAS-HOQGA and QGA

This paper uses the Monte Carlo simulation method for the complete experimental verification across tasks of different scales, and the results are the average values of the algorithm after running 10 times. In addition to comprehensive revenue, the algorithm’s running time is also used as an evaluation indicator for the proposed algorithm. In this paper, the best revenue of each generation will gradually increase and stabilize with the iteration of the algorithm until the revenue no longer changes. When the best revenue no longer changes, it means that the evolution has converged. The shorter the algorithm’s running time to reach the convergence state for the best revenue, the faster the algorithm converges.

**Figure 8:**
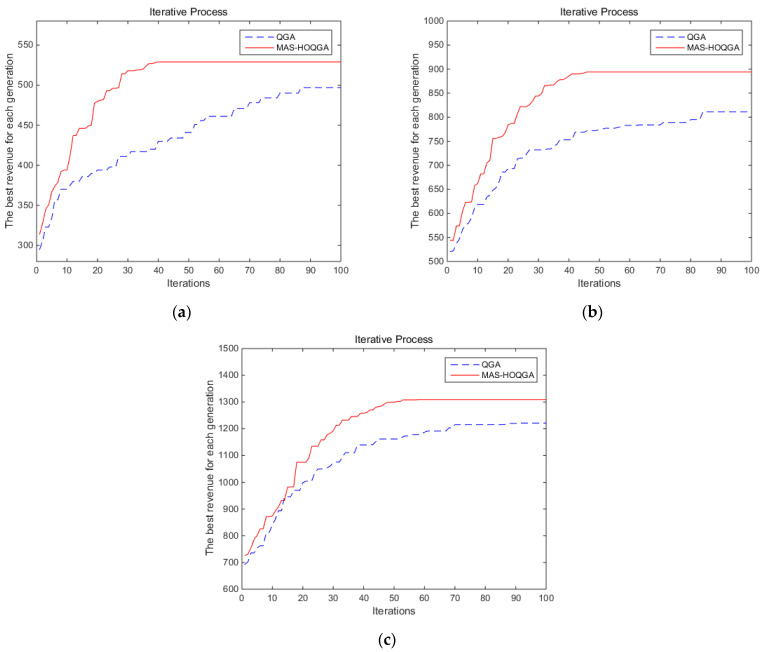
Experimental results of QGA and MAS-HOQGA, with different task scales: (**a**) task scale 226; (**b**) task scale 426; (**c**) task scale 626.

By comparing Figure 8 and Table 2, it can be seen that under the same number of iterations, the MAS-HOQGA can achieve higher revenue, and the convergence speed of the MAS-HOQGA is faster, with fewer iterations corresponding to achieving this optimal revenue. When the task scale is 226, compared with the QGA, the MAS-HOQGA has a 6.44% increase in comprehensive revenue and a 46.9% decrease in algorithm running time. When the task scale is 426, compared with the QGA, the MAS-HOQGA has a comprehensive revenue increase of 10.23% and an algorithm running time decrease of 24.5%. When the task scale is 626, the MAS-HOQGA has a 7.21% increase in comprehensive revenue and a 32.8% decrease in algorithm running time compared to QGA.

#### 4.3.2. Performance Analysis of MAS-HOQGA

1. The impact of probability amplitude adjustment parameter on the MAS-HOQGA

The iterations number was set to 300. The experimental results with different probability amplitude adjustment parameter settings are statistically analyzed and shown in Table 3.

Based on the maximum revenue in the experimental results and the iteration number corresponding to the maximum revenue, it can be concluded that, within a certain range, setting a large probability amplitude adjustment parameter can achieve convergence faster. However, if it is too large, it can easily lead to the algorithm falling into premature convergence, causing local optima. If the parameter is set too small, it can lead to a slow convergence speed. The probability amplitude adjustment parameter adaptive strategy proposed in this paper can reduce time consumption and avoid falling into local optima.

2. Computational complexity analysis of MAS-HOQGA

The calculation speed of the scheduling algorithm is an important factor in evaluating the satellite mission scheduling problems. The faster the algorithm’s calculation speed, the higher the timeliness of the mission scheduling becomes. The speed advantage of quantum computing comes from quantum superposition and quantum entanglement. In this paper, by introducing quantum register operators, the characteristics of quantum superposition and quantum entanglement are utilized to improve the speed of quantum individual measurement and update, effectively reducing the computational complexity of MAS-HOQGA. In this paper, the order of the MAS-HOQGA is set to 2, and theoretically, the time complexity of quantum individual measurement and update can be increased by 50% compared to the traditional QGA. At the same time, the encoding complexity of quantum individuals has not increased, so the running time of the MAS-HOQGA has been greatly improved. However, as the order increases, the encoding complexity of quantum individuals will greatly increase, which will also lead to an increase in the computational complexity of the algorithm. This problem needs to be solved by improving computer performance.

3. The Impact of Task Scale and Constraints on MAS-HOQGA

Through theoretical analysis, it can be determined that the MAS-HOQGA has good scalability in terms of task scale and scheduling model constraints. Firstly, the task scale determines the length of the quantum chromosome, which will only affect the computational complexity of the MAS-HOQGA, but it will not affect the solution methodology proposed in this paper. Secondly, increasing the constraints of the scheduling model will affect both the running time of the scheduling algorithm and the number of executable tasks in the final solution, but it will not affect the solution methodology of the MAS-HOQGA.

## 5. Conclusions

The agile remote sensing satellite scheduling problem for large-scale tasks, considering complex constraints and a large-scale solution space, is studied in this paper, in which the solving speed and comprehensive revenue are optimized simultaneously. In this work, we introduced the quantum register operator and innovatively proposed a multi-adaptive strategies-based higher-order quantum genetic algorithm. Computational experiments prove that when the MAS-HOQGA is applied to satellite scheduling problems, compared with the QGA, the MAS-HOQGA speeds up the mission scheduling algorithm’s running time, prevents the algorithm from falling into local optima, and thus results in higher comprehensive revenue. The content studied in this paper is applicable to satellite scheduling problems based on large-scale tasks and can also provide ideas for solving multi-satellite and multi-task scheduling problems in large-scale constellations.

It is worthwhile to explore the influence of the order of quantum registers on the performance of the algorithm in high-order quantum genetic algorithms. In addition, in view of the future multi-satellite coordination problem, addressing how to improve the current algorithm to adapt to the multi-satellite coordination scenario will become the focus of future research. We hope to combine the improved MAS-HOQGA with appropriate multi satellite collaboration strategies to solve the satellite scheduling problems in both large-scale satellites and large-scale tasks scenarios, aiming to solve the large-scale agile remote sensing satellite mission scheduling problem in engineering practice.

## Figures and Tables

**Table 1:** Orbit parameter.

Orbit Parameter	Set Value
Epoch Time (UTCG)	21 March 2023 00:00:00.000
Semi-major Axis (km)	6862.92
Eccentricity	1.89 × 10^−16^
Inclination (deg)	97.6000
RAAN (deg)	44.5853
Arg of Perigee (deg)	0.0000
True Anomaly (deg)	0.0000

**Table 2:** The experimental results obtained by QGA and MAS-HOQGA.

Algorithm\Task Scales	226		426		626	
	Rev	Time/s	Rev	Time/s	Rev	Time/s
QGA	497	56.62	811	89.83	1221	148.95
MAS-HOQGA	529	30.05	894	67.79	1309	100.05

**Table 3:** The experimental results for different probability amplitude adjustment parameters.

Parameter Settings	0.99	0.985	0.98	0.975	0.97	0.95	Adaptive Value
Rev	1309	1309	1309	1299	1232	1209	1309
Iteration Number Corresponding to the Maximum Revenue	391	284	217	175	155	103	162
Time/s	364.09	347.11	319.38	301.28	285.93	284.91	286.65

## Data Availability

Data are contained within the article.

## References

[1] Neeck S.P., Magner T.J., Paules G.E. (2005). NASA’s small satellite missions for Earth observation. Acta Astronaut..

[2] Wang X., Wu G., Xing L., Pedrycz W. (2021). Agile earth observation satellite scheduling over 20 years: Formulations, methods, and future directions. IEEE Syst. J..

[3] Agn J.C., Bataille N., Blumstein D., Bensana E., Verfaillie G. (2007). Exact and approximate methods for the daily management of an earth observation satellite. RAIRO-Oper. Res..

[4] Bensana E., Lemaitre M., Verfaillie G. (1999). Earth observation satellite management. Constraints.

[5] Lemaître M., Verfaillie G., Jouhaud F., Lachiver J., Bataille N. How to manage the new generation of agile earth observation satellites. Proceedings of the International Symposium on Artificial Intelligence, Robotics and Automation in Space.

[6] Liu H., Pang L., Zhang L., Huo X., Luan J.Y., Lan K.X., Jing C.F., Li W. A cooperation earth observation model of SAR satellite and optical remote sensing satellite. Proceedings of the 2014 IEEE Geoscience and Remote Sensing Symposium.

[7] Abramson M., Carter D., Kolitz S., Ricard M., Scheidler P. Real-time optimized earth observation autonomous planning. Proceedings of the NASA Earth Science Technology Conference.

[8] Bianchessi N., Righini G. (2008). Planning and scheduling algorithms for the COSMO-SkyMed constellation. Aerosp. Sci. Technol..

[9] Florio S.D. Performances optimization of remote sensing satellite constellations: A heuristic method. Proceedings of the 5th International Workshop on Planning and Scheduling for Space.

[10] Lemaître M., Verfaillie G., Jouhaud F., Lachiver J.M., Bataille N. (2002). Selecting and scheduling observations of agile satellites. Aerosp. Sci. Technol..

[11] He R., Gao P., Bai B.C., Li J., Yao F., Xing L.N. (2011). Models, algorithms and applications to the mission planning system of imaging satellites. Syst. Eng. Theory Pract..

[12] He C., Zhu X.M., Qiu D.S. (2012). Cooperative scheduling method of multi-satellites for imaging reconnaissance in emergency condition. J. Syst. Eng. Electron..

[13] Peng G., Song G., He Y., Yu J., Vansteenwegen P. (2002). Solving the agile earth observation satellite scheduling problem with time-dependent transition times. IEEE Trans. Syst. Man. Cybern..

[14] Xu R., Chen H., Liang X., Wang H. (2016). Priority-based constructive algorithms for scheduling agile earth observation satellites with total priority maximization. Expert. Syst. Appl..

[15] He L., Liu X.L., Laporte G., Chen Y.W., Chen Y.G. (2018). An improved adaptive large neighborhood search algorithm for multiple agile satellites scheduling. Comput. Oper. Res..

[16] She Y., Li S., Zhao Y. (2018). Onboard mission planning for agile satellite using modified mixed-integer linear programming. Aerosp. Sci. Technol..

[17] Du B., Li S. (2019). A new multi-satellite autonomous mission allocation and planning method. Acta Astronaut..

[18] Qiu D., Guo H., He C., Wu G. (2013). Intensive task scheduling method for multi-agile imaging satellites. Acta Aeronaut. Astronaut. Sin..

[19] Li Y., Wang R., Xu M. (2014). Rescheduling of observing spacecraft using fuzzy neural network and ant colony algorithm. Chin. J. Aeronaut..

[20] Lu Z., Shen X., Li D., Chen Y. (2022). Integrated Imaging Mission Planning Modeling Method for Multi-Type Targets for Super-Agile Earth Observation Satellite. IEEE J-Stars..

[21] Han P., He Z., Geng Y., Guo Y., Li C., Zhao G. Mission planning for agile earth observing satellite based on genetic algorithm. Proceedings of the 2019 Chinese Control Conference.

[22] Li Y., Xu M., Wang R. Scheduling observations of agile satellites with combined genetic algorithm. Proceedings of the Third International Conference on Natural Computation.

[23] Chen C., Xing L., Tan Y. (2010). Improved genetic algorithm for cooperating multi air vehicle mission planning. Ordnance Ind. Autom..

[24] Zheng Z., Guo J., Gill E. (2018). Onboard autonomous mission re-planning for multi-satellite system. Acta Astronaut..

[25] Han C., Bai S., Zhang S., Wang X. (2019). Visibility optimization of satellite constellations using a hybrid method. Acta Astronaut..

[26] Du Y., Liao K.F., Ouyang S., Li J.J., Huang G.J. (2020). Time and Aperture Resource Allocation Strategy for Multitarget ISAR Imaging in a Radar Network. IEEE Sens. J..

[27] Zhang J., Xing L. (2022). An improved genetic algorithm for the integrated satellite imaging and data transmission scheduling problem. Comput. Oper. Res..

[28] Dilkina B., Havens B. (2005). Agile Satellite Scheduling via Permutation Search with Constraint Propagation.

[29] Globus A., Crawford J., Lohn J., Pryor A. A comparison of techniques for scheduling earth observing satellites. Proceedings of the National Conference on Artificial Intelligence.

[30] Han C., Gu Y., Wu G., Wang X. (2023). Simulated Annealing-Based Heuristic for Multiple Agile Satellites Scheduling Under Cloud Coverage Uncertainty. IEEE T Syst. Man Cybern. Syst..

[31] Bianchessi N., Cordeau J.F., Desrosiers J., Laporte G., Raymond V. (2007). A heuristic for the multi-satellite, multi-orbit and multi-user management of Earth observation satellites. Eur. J. Oper. Res..

[32] Habet D., Vasquez M. Saturated and consistent neighborhood for selecting and scheduling photographs of agile earth observing satellite. Proceedings of the 5th Metaheuristics International Conference.

[33] Zuo C., Wang H. (2010). Research on scheduling of earth observing satellites based on taboo search algorithm. Comput. Eng. Appl..

[34] Habet D., Vasquez M., Vimont Y. (2010). Bounding the optimum for the problem of scheduling the photographs of an agile earth observing satellite. Comput. Optim. Appl..

[35] Zheng Q., Yue H., Liu D., Jia X. (2023). A Scheduling Method of Using Multiple SAR Satellites to Observe a Large Area. Sensors.

[36] Povéda G., Regnier-Coudert O., Teichteil-Königsbuch F., Dupont G., Arnold A., Guerra J., Picard M. Evolutionary approaches to dynamic earth observation satellites mission planning under uncertainty. Proceedings of the Genetic and Evolutionary Computation Conference.

[37] Liu D., Chang S., Deng Y., He Z., Wang F., Zhang Z., Han C., Yu C. (2024). A Novel Spaceborne SAR Constellation Scheduling Algorithm for Sea Surface Moving Target Search Tasks. IEEE J-Stars..

[38] Liu X.W., Zhang Q., Luo Y., Lu X., Dong C. (2021). Radar Network Time Scheduling for Multi-Target ISAR Task with Game Theory and Multiagent Reinforcement Learning. IEEE Sens. J..

[39] Li D.L., Wang H.J., Yang Z., Gu Y.F., Shen S. (2021). An Online Distributed Satellite Cooperative Observation Scheduling Algorithm Based on Multiagent Deep Reinforcement Learning. IEEE Geosci. Remote Sens. Lett..

[40] Liu Y., Chen Q., Li C., Wang F. (2021). Mission planning for Earth observation satellite with competitive learning strategy. Aerosp. Sci. Technol..

[41] Chen J., Chen M., Wen J., He L., Liu X. (2022). A heuristic construction neural network method for the time-dependent agile earth observation satellite scheduling problem. Mathematics.

[42] Du Y., Wang T., Xin B., Wang L., Xing L. (2020). A data-driven parallel scheduling approach for multiple agile earth observation satellites. IEEE Trans. Evol. Comput..

[43] He Y., Xing L., Chen Y., Pedrycz W., Wu G. (2022). A generic Markov decision process model and reinforcement learning method for scheduling agile earth observation satellites. IEEE Trans. Syst. Man. Cybern..

[44] Xu Z., Wang H., Xing C., Tao T., Mao J., Liu Y. (2023). Physics guided wavelet convolutional neural network for wind-induced vibration modeling with application to structural dynamic reliability analysis. Eng. Struct..

[45] Narayanan A., Moore M. Quantum-inspired genetic algorithms. Proceedings of the IEEE International Conference on Evolutionary Computation.

[46] Da Silveira L.R., Tanscheit R., Vellasco M.M.B.R. (2017). Quantum inspired evolutionary algorithm for ordering problems. Expert. Syst. Appl..

[47] Nowotniak R., Kucharski J. Higher-order quantum-inspired genetic algorithms. Proceedings of the 2014 Federated Conference on Computer Science and Information Systems.

